# Conceptual issues in relation to the design, implementation and evaluation of interventions

**DOI:** 10.25646/6501

**Published:** 2020-06-04

**Authors:** Eva A. Rehfuess

**Affiliations:** 1 Ludwig Maximilian University of Munich Institute for Medical Information Processing, Biometry, and Epidemiology; 2 Ludwig Maximilian University of Munich Pettenkofer School of Public Health

The conceptualization of public health interventions as events in complex systems represents a key starting point for this contribution [1]. Importantly, the design, evaluation and implementation of interventions rarely occur in a linear sequence but tend to involve iterations in a cyclical fashion. Generating research evidence across these stages ideally involves different public health stakeholders in order to generate policy-relevant evidence.

This contribution focuses on two aspects of relevance across intervention design, evaluation and implementation: the need to ask a range of questions and to consider a range of evidence beyond evidence of effectiveness; and the recognition that public health interventions depend on and interact with their context. These two aspects are introduced using examples of interventions targeting individuals, populations and systems. As the ‘rhetoric urging complex systems approaches to public health is only rarely operationalised in ways that generate relevant evidence of effective policies’ [2], the contribution also presents selected tools to address these challenges in primary research, evidence synthesis and decision-making.

Whether a public health measure – for example a behavioural intervention to promote healthy eating – is introduced by decision-makers and adopted and maintained by target populations is influenced by many factors. Logic models, ‘a graphic description of a system … designed to identify important elements and relationships within that system …’, are a tool for being explicit about the intervention and its components and for thinking through other important factors and the interactions between them [3, 4]. With respect to evidence needs for decision-making, evidence-to-decision (EtD) frameworks propose a set of criteria, thereby making value assumptions explicit. The WHO-INTEGRATE EtD framework, firmly rooted in World Health Organization (WHO) norms and values, proposes six substantive criteria – balance of health benefits and harms, human rights and socio-cultural acceptability, health equity, equality and non-discrimination, societal implications, financial and economic considerations and feasibility and health system considerations – as well as the meta-criterion quality of evidence [5].

Context matters – as illustrated by the health system intervention to deliver antenatal corticosteroids showing largely positive impacts in high-income settings but substantial harm in low-income settings. Context is defined as ‘… any feature of the circumstances in which an intervention is implemented that may interact with the intervention to produce variation in outcomes’ [6]. The Context and Implementation of Complex Interventions (CICI) framework represents a tool for reflecting on context in a comprehensive manner and for considering how contextual domains – i.e. geographical, epidemiological, socio-economic, socio-cultural, legal, political, ethical – might influence how an intervention works (or not), and how intervention impacts vary [7]. This has important implications for the transferability of interventions from one context to another, with guidance on how to adapt and re-evaluate interventions currently being developed [8].

This contribution has focused on only two conceptual issues affecting the design, evaluation and implementation of interventions – the broad range of evidence needs and the importance of context. Taking a complexity perspective and its implications seriously is likely to require ‘far reaching changes to the way population health intervention research is funded, conducted and published’ [6].
